# Genetic Basis of Tobacco Use Disorder

**DOI:** 10.3390/genes17091064

**Published:** 2026-09-02

**Authors:** Guillermo Gervasini

**Affiliations:** 1 Department of Medical-Surgical Therapeutics, Faculty of Medicine, University of Extremadura, 06006 Badajoz, Spain; ggervasi@unex.es; 2 Institute of Molecular Pathology Biomarkers, University of Extremadura, 06006 Badajoz, Spain

**Keywords:** smoking, addiction, genetics, cardiovascular risk

## Abstract

Tobacco use disorder (TUD) is a complex multifactorial condition resulting from the interplay between nicotine-induced neurobiological adaptations, behavioral and learning processes, environmental influences, and individual genetic susceptibility. Genetic research has progressively evolved from twin and family studies through candidate-gene approaches to large-scale genome-wide association studies (GWAS), substantially improving our understanding of the genetic architecture of tobacco use and nicotine dependence. This review summarizes the current evidence on the genetic basis of TUD, with particular emphasis on major biological pathways. We also discuss the limitations of early candidate-gene studies and the paradigm shift introduced by large GWAS and meta-analyses, and polygenic risk scores, which indicate that tobacco use and nicotine dependence have a highly polygenic and pleiotropic architecture. Finally, we review the potential clinical applications of genetic information in smoking-cessation treatment, while highlighting current limitations in clinical translation. In the future, it is clear that a multidisciplinary approach—combining genetics, clinical practice, and social sciences—will be necessary to transform tobacco use management into a precision-based model and reduce its impact on public health.

## 1. Introduction

Tobacco smoking remains one of the greatest public health challenges worldwide. According to the World Health Organization, approximately 1.3 billion people smoke globally, and tobacco use is responsible for an estimated 8 million deaths each year, including around 1.3 million among non-smokers exposed to second-hand smoke [1]. Smoking promotes oxidative stress and proatherogenic processes that double the 10-year risk of fatal cardiovascular events compared with non-smokers, whereas smoking cessation at an early age can reduce the excess risk of death by up to 90% [2].

Tobacco use disorder (TUD) is a complex multifactorial condition characterized by compulsive tobacco use, difficulty controlling consumption, continued use despite adverse consequences, and withdrawal symptoms upon cessation. Nicotine is the principal addictive component of tobacco and acts primarily through neuronal nicotinic acetylcholine receptors (nAChRs), which are expressed across multiple interconnected brain circuits [3,4]. Nicotine exposure modulates dopaminergic, glutamatergic, GABAergic, and other neurotransmitter systems, thereby influencing reinforcement, motivation, learning, and behavioral adaptation. Repeated exposure produces neuroadaptations that contribute to tolerance and dependence, while cessation produces a characteristic withdrawal syndrome [5]. Importantly, nicotine dependence cannot be reduced to its acute rewarding effects: conditioned environmental cues, incentive salience, stress, negative reinforcement, and persistent changes in learning and habit-related circuits all contribute to the maintenance of tobacco use and relapse. Consequently, current clinical practice guidelines recommend not only pharmacological treatments but also cognitive behavioral therapy, either alone or in combination with medication, to maximize smoking cessation success [6]. Thus, TUD emerges from the dynamic interaction between nicotine-induced neurobiological adaptations, behavioral and learning processes, environmental influences, and individual genetic susceptibility. The contribution of the latter will be examined in detail in the following sections.

## 2. Evidence for the Influence of Genetics on Tobacco Use Disorder

### 2.1. Twin Studies

**Figure 1:**
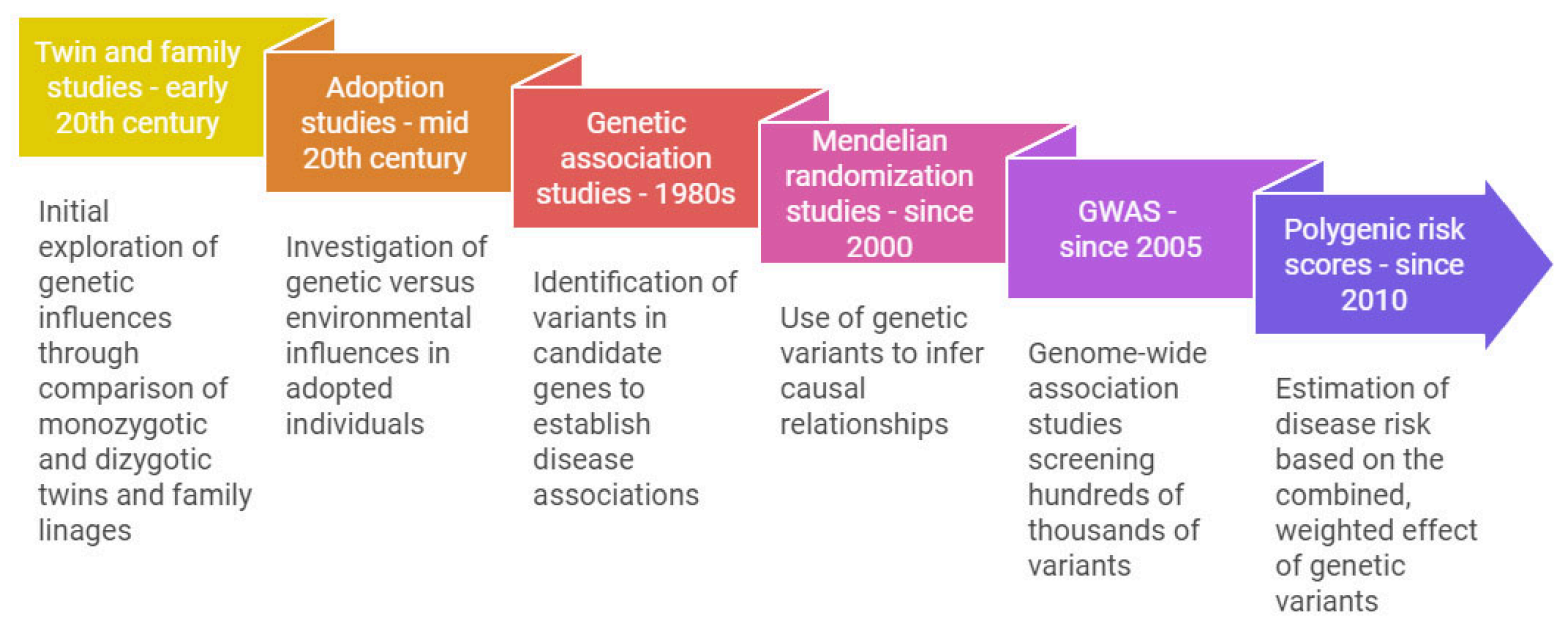
Evolution of the genetic research methods used in the field of tobacco use disorder.

The relationship between genetics and TUD has been supported by an increasing body of evidence (Figure 1), with twin studies providing some of the earliest and most influential findings [7]. These studies compare monozygotic twins, who share virtually all of their genetic material, with dizygotic twins, who share approximately half. If a given phenotype—for example, smoking behavior—is more concordant among monozygotic than dizygotic twins, this strongly suggests the presence of a genetic contribution.

The first twin studies demonstrated that several smoking-related traits—including persistence of smoking, success in smoking cessation, age at smoking initiation, and the response to first nicotine exposure—are influenced by genetic factors [8,9,10,11]. Since these characteristics predict both vulnerability to tobacco use and patterns of nicotine dependence, they provide compelling evidence that inherited biological factors contribute substantially to TUD. A subsequent meta-analysis of twin studies confirmed that both genetic and environmental factors influence smoking initiation and persistence, demonstrating that tobacco dependence is not merely a behavioral phenomenon but is strongly shaped by biological predisposition [12].

Although twin studies, together with family and adoption studies, have provided invaluable insights into the heritability of TUD, they cannot identify the specific genetic variants responsible for these effects. This limitation has been largely overcome by genome-wide association studies (GWAS), which have identified numerous loci associated with smoking-related phenotypes.

### 2.2. Genome-Wide Association Studies

Genome-wide association studies compare the allele frequencies of hundreds of thousands—or even millions—of genetic variants between individuals displaying different phenotypes, such as smokers and non-smokers. These studies have become the principal approach for establishing robust associations between genetic variation and tobacco-related behaviors.

Early GWAS focused primarily on common variants, defined as those with allele frequencies greater than 5% [13,14]. Advances in genotype imputation and the increasing availability of large international cohorts have greatly improved statistical power and enabled large-scale meta-analyses, leading to the identification of numerous genomic regions associated with smoking initiation, smoking cessation, and smoking intensity. Among these, the 15q25 locus has consistently emerged as the strongest genetic determinant of TUD. Within this region, the regulatory variant rs55853698, located near the *CHRNA5* gene, which encodes a subunit of the nAChR, has shown one of the largest effects on smoking behavior [15]. The 15q25 region has likewise been identified as the most significant locus in several independent GWAS [16] and in meta-analyses including individuals of African ancestry [17]. In Asian populations, additional susceptibility regions have been identified, including 7q31.1 [18], as well as several variants associated with age at smoking initiation and cigarettes smoked per day [19]. In European populations, a GWAS conducted in Dutch siblings identified loci on chromosomes 5, 14, and 22 associated with age at first cigarette. The strongest signal was located on chromosome 5 within a region containing the dopamine D1 receptor (*DRD1*) gene, a key component of the endogenous reward system [20]. More recently, two meta-analyses by Erzurumluoglu et al. and Xu et al., including over 600,000 and 800,000 individuals, respectively, identified an additional 55 loci associated with smoking-related behaviors, further expanding our understanding of the genetic architecture of TUD [21,22].

As shown above, ancestry is an important consideration when interpreting genetic findings in TUD. Although smoking-related GWAS have increased substantially in size, they have historically been dominated by individuals of European ancestry. For example, the large-scale study by Saunders et al. [23] included 3.4 million individuals, but only 21% were of non-European ancestry, highlighting the persistent imbalance in genetic studies of tobacco use. Differences in linkage disequilibrium (LD) patterns between populations can influence the ability to detect and fine-map causal variants, as SNPs in LD with the causal variant in one population may be less strongly correlated in another. Importantly, these differences can also affect the transferability of PRS. It has been shown that tobacco-use PRS may perform well within European-ancestry populations while having limited predictive accuracy when transferred across ancestries [23]. This issue is particularly relevant for admixed populations, which remain substantially underrepresented in genetic studies and present complex patterns of local ancestry and LD that are not adequately captured by the present scores.

Despite these remarkable advances, the proportion of phenotypic variance explained by GWAS-identified variants remains substantially lower than heritability estimates derived from twin studies, which suggests a polygenic architecture discussed in the next section. Nevertheless, even the strongest genetic associations should not overshadow the importance of environmental influences. Vink and colleagues demonstrated that environmental factors exert a major influence on smoking frequency and relapse risk, reinforcing the concept that TUD is a multifactorial disorder in which genetic susceptibility interacts continuously with social and behavioral determinants to shape individual risk [24].

### 2.3. Polygenic Risk Scores

Polygenic risk scores (PRS) are quantitative measures of an individual’s genetic predisposition to a complex trait, calculated by combining the effects of multiple genetic variants. Unlike candidate-gene approaches, PRS aggregate the small effects of numerous variants across the genome and therefore provide a more comprehensive measure of genetic liability. This approach is particularly relevant to tobacco use and nicotine dependence, which have a highly polygenic genetic architecture [25].

Several studies have demonstrated associations between PRS and smoking-related phenotypes. PRS have been associated with smoking initiation, cigarettes per day, smoking cessation, or nicotine dependence [25,26,27]. The expansion of GWAS sample sizes has considerably improved their predictive performance. For example, Saunders et al. conducted a large multi-ancestry GWAS involving approximately 3.4 million individuals and demonstrated the predictive utility of tobacco-use PRS, while also highlighting their reduced transferability across ancestral populations [23].

Overall, current evidence supports the view that tobacco use and nicotine dependence are highly polygenic traits, in which susceptibility is influenced by the cumulative effects of numerous genetic variants, each generally exerting a small effect, rather than by a limited number of major susceptibility genes [28]. Moreover, the risk for TUD seems to be not only polygenic, but also pleiotropic, as genetic variants associated with nicotine dependence also tend to be associated with other behavioral, psychiatric, or substance-use traits [28]. Thus, although specific loci such as the *CHRNA5–CHRNA3–CHRNB4* cluster have important biological effects, they represent only part of a much broader polygenic architecture.

## 3. Major Genetic Pathways Involved in Tobacco Use Disorder

**Figure 2:**
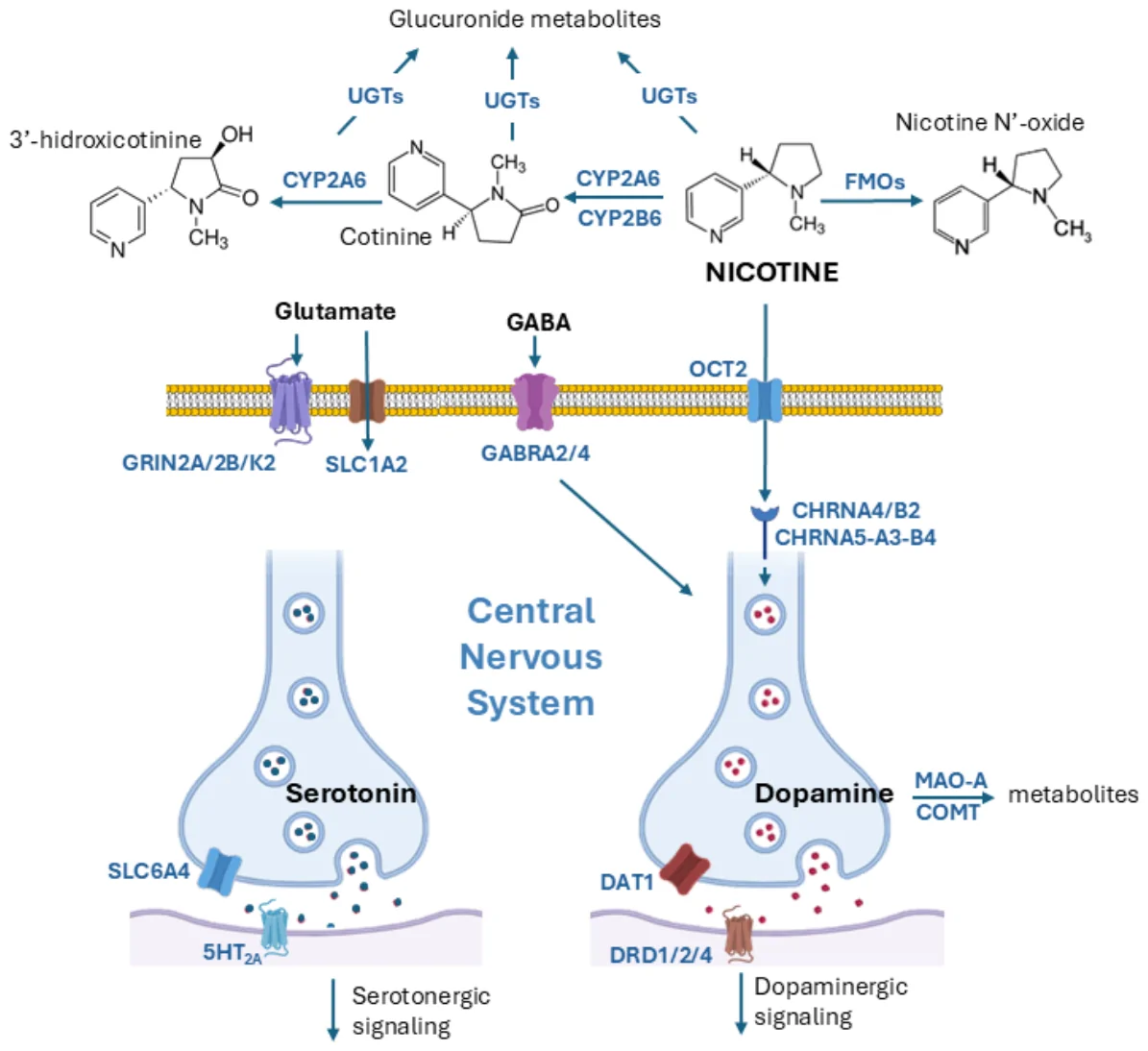
Overview of the major genetic pathways involved in TUD. 5HT2A, serotonin 2A receptor; COMT, catechol-O-methyltransferase; CHRNA4/B2 and CHRNA5-A3-B4, nicotinic acetylcholine receptors; CYP2A6 and CYP2B6, cytochrome P450 2A6 and 2B6; DAT1, dopamine transporter; DRD2/3/4, dopamine receptors 2, 3, and 4; FMOs, flavin-containing monooxygenases; GRIN2A/2B/K2, NMDA glutamate receptor subunits; GABRA2/4, GABA receptor subunits; MAO-A, monoamine oxidase A; OCT2, nicotine and varenicline transporter; SLC1A2, glutamate transporter; SLC6A4, serotonin transporter; UGTs, uridine diphosphate glucuronosyltransferases.

TUD results from the interaction of multiple genetic pathways (Figure 2, Table 1), each contributing to different aspects of tobacco use, dependence, and abstinence. The accumulated evidence highlights that a thorough understanding of these pathways is not only essential for elucidating the pathophysiology of TUD, but also for designing personalized interventions that maximize treatment efficacy, as discussed in later sections.

### 3.1. Nicotinic Acetylcholine Receptors

Among the genes identified, those encoding nAChR subunits have received particular attention in recent years. The *CHRNA5-CHRNA3-CHRNB4* gene cluster, located on chromosome 15, has been consistently associated with smoking susceptibility and nicotine dependence. A study by Thorgeirsson et al. [13] identified specific variants within this locus that significantly increase the risk of developing TUD. Variant rs16969968 and other variants in linkage disequilibrium produced the strongest association signal within this cluster [29]. Other variants within this cluster have also been associated with different smoking-related characteristics, including smoking initiation, smoking intensity, nicotine dependence, and persistence of tobacco use [29]. In addition, *CHRNB2* has been associated with susceptibility to smoking initiation [30], and a rare variant in *CHRNA4* appears to reduce receptor sensitivity and increase the risk of nicotine addiction [31].

### 3.2. Dopaminergic System

The dopaminergic system plays a crucial role in addiction, as it mediates the rewarding effects that reinforce nicotine consumption. Consequently, genes involved in this pathway have been the subject of numerous association studies [32]. For example, the *DRD2* gene, which encodes the dopamine D2 receptor, has been associated with susceptibility to smoking. Specifically, the Taq1A variant (rs1800497) has been linked to a lower density of D2 receptors [33], which could interfere with the dopaminergic reward system. A meta-analysis including 11,000 smokers concluded that carriers of this variant were more likely to achieve successful smoking cessation during treatment [34]. In addition, a multi-ancestry meta-analysis by Saunders et al. found an association between variants in the neurturin (*NRTN*) gene, encoding a type of glial cell line involved in the survival of dopamine neurons, and cigarettes smoked per day. The same study revealed that another gene implicated in the modulation of dopaminergic neurotransmission, *PAK6*, was also strongly associated with the number of cigarettes smoked [23]. Variants in the dopamine transporter *DAT1* (*SLC6A3*) gene, which affect dopamine reuptake and consequently dopamine levels within the synaptic cleft, have also been linked to tobacco dependence. Thus, Tiili et al. reported that a VNTR polymorphism in this gene may reduce the risk of smoking initiation as a consequence of decreased dopamine availability [35]. Regarding dopamine metabolism, McKinney et al. found that variability in the genes encoding monoamine oxidase (MAO)-A and dopamine-β-hydroxylase influences cigarette consumption [36]. Although there is strong evidence supporting the involvement of dopaminergic genetics in TUD, it should be emphasized that the results concerning these genes have not yet been consistently replicated. In most cases, this is because the genetic association studies conducted lacked sufficient sample size to detect the effects of common genetic variants on complex phenotypes such as smoking behavior, a limitation that has largely been overcome by GWAS.

### 3.3. Serotonergic System

The evidence on the serotonergic system is far less significant. The T102C variant in the 5-HT2A serotonin receptor has been associated with maintenance of the smoking habit [37], although further studies are needed to confirm these findings. Similarly, the *SLC6A4* gene, which encodes the serotonin transporter, has also been studied in this regard, but results are far from being conclusive.

### 3.4. Cytochrome P450

Within the cytochrome P450 (CYP450) enzyme system, CYP2A6 is responsible for metabolizing approximately 80% of nicotine in the liver, converting it into cotinine, and subsequently catalyzing the biotransformation of cotinine into 3-hydroxycotinine [38] (Figure 2), illustrating its importance in studies conducted to date. Variants in the *CYP2A6* gene have been associated with numerous characteristics related to smoking behavior [39]. For example, slow metabolizers have a greater risk of developing nicotine dependence, but they also tend to smoke fewer cigarettes and are more likely to quit smoking spontaneously, probably because they experience less severe withdrawal symptoms [40,41]. More than 40 *CYP2A6* variants affecting enzymatic activity have been described, and this locus contains many of the variants identified as significant in GWAS conducted in smokers [42].

CYP2B6 is the second most active enzyme involved in nicotine metabolism. This gene is also highly polymorphic and is expressed at significant levels in the brain, where it may play a major role in the local metabolism of nicotine [43]. However, its genetic variability appears to be clinically relevant mainly in relation to the efficacy of bupropion treatment, since this drug is metabolized by CYP2B6 (see below).

### 3.5. GABAergic and Glutamatergic Systems

The GABAergic system, which plays a key role in regulating neuronal excitability, also participates in TUD, probably through interactions with the dopaminergic system. Accordingly, variants in the *GABRA4* and *GABRA2* genes, which encode GABA receptor subunits, have been associated with scores on the Fagerström Test for Nicotine Dependence [44].

The glutamatergic system contributes through NMDA receptors, which are involved in synaptic plasticity and associative memory, processes that are essential for the consolidation of addictive behaviors [45]. Consistent with this hypothesis, the rs4354668 variant in the glutamate transporter *SLC1A2* has recently been associated with an increased risk of drug use [46]. Likewise, several NMDA glutamate receptor subunits (*GRIN2A*/*2B*/*K2*) and *SLC1A2* were identified in a GWAS investigating smoking-related behavior [47], further supporting the importance of this pathway in TUD.

### 3.6. Other Candidate Pathways

There is also evidence suggesting that TUD is modulated by variants in genes involved in other biological pathways, including different flavin-containing monooxygenases (FMO) [48,49] and UDP-glucuronosyltransferases (UGT2B10 and UGT2B17) [48,50], which are involved in nicotine metabolism. In addition, genes belonging to the neuregulin signaling pathway have been implicated [51], as they are important in the well-established relationship between schizophrenia and nicotine dependence. Furthermore, TUD is also influenced by epigenetic modifications. Furthermore, epigenetic mechanisms may contribute to the relationship between genetic susceptibility, tobacco exposure, and tobacco-related disease. It is important, however, to distinguish pre-existing epigenetic profiles that may contribute to individual susceptibility to TUD from epigenetic alterations acquired as a consequence of tobacco exposure. For example, tobacco smoking induces persistent changes in DNA methylation at genomic regions such as *AHRR* and *GPR15*, which have been associated with cumulative smoking exposure and smoking-related diseases [52]. These exposure-related epigenetic changes should therefore not necessarily be interpreted as pre-existing determinants of tobacco use, but rather as potential molecular consequences or mediators of tobacco exposure.

The evidence discussed above illustrates the important contribution of candidate-gene studies to the early understanding of the genetic basis of TUD. However, their interpretation should be considered in the context of the major methodological shift introduced by GWAS. Candidate-gene studies were generally based on biologically plausible hypotheses and focused on a limited number of variants, but many reported associations have shown inconsistent replication when tested in larger and more diverse populations, e.g., studies on *DRD4* or *SLC6A4*. In contrast, GWAS provide an unbiased, genome-wide approach and, particularly when combined through large-scale meta-analyses, have substantially increased statistical power and yielded more robust and reproducible associations [14,15,16,22,23,25]. Importantly, GWAS findings have also shifted the conceptual framework from a model centered on a small number of susceptibility genes toward a complex and highly polygenic architecture, in which numerous variants of individually small effect contribute to TUD. Thus, while candidate-gene studies remain valuable for understanding specific biological pathways and generating mechanistic hypotheses, the strongest current evidence regarding the genetic architecture of TUD derives from large-scale GWAS and meta-analyses.

**Table 1:** Summary of associations of genetic variability in the major pathways involved in tobacco use disorder.

Reference	Pathway	Gene(s) and Variant(s)	Comment
Thorgeirsson et al., 2008 [13]	Nicotinic acetylcholine receptors	*CHRNA5-CHRNA3-CHRNB4* (15q25 locus)	First GWAS to identify the 15q25 cluster as the major genetic determinant of nicotine dependence.
Lassi et al., 2016 [29]	Nicotinic acetylcholine receptors	*CHRNA5* rs16969968	Review identifying rs16969968 as the most relevant variant associated with smoking intensity and nicotine dependence.
Greenbaum et al., 2006 [30]	Nicotinic acetylcholine receptors	*CHRNB2* CACTA haplotype	Associated with reduced susceptibility to smoking initiation.
Thorgeirsson et al., 2016 [31]	Nicotinic acetylcholine receptors	*CHRNA4* rs56175056	This variant increases the risk of nicotine addiction.
Munafo et al., 2004 [32]	Dopaminergic system	*DRD2*, *DRD4*, *SLC6A3*, *MAO-A*, *DBH*	Systematic review of the association between variability in dopaminergic genes and smoking.
Ma et al., 2015 [34]	Dopaminergic system	*DRD2* rs1800497 (Taq1A)	Meta-analysis associating the variant with a higher likelihood of successful smoking cessation.
Saunders et al., 2022 [23]	Dopaminergic system	NRTN, several variants	Association with number of cigarettes smoked according to a large-scale meta-analysis
Saunders et al., 2022 [23]	Dopaminergic system	PAK6, several variants	Association with number of cigarettes smoked according to a large-scale meta-analysis
Tiili et al., 2020 [35]	Dopaminergic system	*SLC6A3* (DAT1) VNTR	The variant reduces the risk of smoking initiation through reduced dopamine availability.
McKinney et al., 2000 [36]	Dopaminergic system	*MAO-A* rs1137070 and *DBH* rs1108580	Variants associated with the number of cigarettes smoked.
do Prado-Lima et al., 2004 [37]	Serotonergic system	*HTR2A* rs6313	Associated with maintenance of the smoking habit.
Malaiyandi et al., 2006 [40]	Nicotine metabolism (CYP450)	*CYP2A6* (slow-metabolizer alleles)	Slow metabolizers show lower cigarette consumption and a higher likelihood of spontaneous smoking cessation.
Agrawal et al., 2009 [44]	GABAergic system	20 variants in *GABRA2* and *GABRA4*	Associated with Fagerström Test scores for nicotine dependence.
Dawidowski et al., 2024 [46]	Glutamatergic system	*SLC1A2* rs4354668	The variant increases the risk of drug use and possibly smoking.
Vink et al., 2009 [47]	Glutamatergic system	*GRIN2A*, *GRIN2B*, *GRIK2*, *SLC1A2*	GWAS identifying glutamatergic genes associated with age at smoking initiation.
Zhang et al., 2017 [49]; Pérez-Páramo et al., 2023 [48]	Nicotine metabolism	*FMO1* rs6674596	Variant associated with nicotine dependence in European populations.
Ware et al., 2016 [50]; Pérez-Páramo et al., 2023 [48]	Nicotine metabolism	*UGT2B10* rs835316, rs2942857; *UGT2B17* deletion	Variants associated with cotinine metabolism.
Loukola et al., 2014 [51]	Neuregulin pathway	*ERBB4* rs7562566	Possible relationship between nicotine dependence and schizophrenia.
Zeilinger et al., 2013 [52]	Epigenetics	*AHRR* cg05575921	Smoking induces DNA methylation changes associated with tobacco use and smoking-related diseases.

## 4. Genetics in Therapeutic Strategies for Tobacco Use Disorder

The accumulated knowledge on the genetic basis of TUD has the potential to drive the development of personalized medicine strategies for tobacco dependence, with the aim of improving the efficacy of interventions, minimizing adverse effects, and creating opportunities for early prevention. Simply informing a smoker that their genetic profile may place them at increased risk of developing tobacco-related diseases may increase their motivation to quit smoking [53].

As described before, the clinical implementation of PRS has become a major area of research in numerous diseases [54]. Accordingly, several of these scores have been developed in relation to TUD and may help predict the likelihood of smoking initiation and nicotine dependence [27,55,56,57]. These tools could therefore help target preventive interventions towards high-risk populations. One example is a study involving more than 300,000 participants that proposed the use of these scores to identify high-risk smokers and optimize lung cancer screening strategies [58], although there are also data questioning the cost-effectiveness of such programs [59]. The usefulness of these PRS has also been evaluated in smoking cessation therapies, and several studies and meta-analyses suggest that they may be useful for predicting the success of treatment [60,61].

On the other hand, pharmacological treatment for TUD, including agents such as varenicline, bupropion, cytisinicline, and nicotine replacement therapies, could also benefit substantially from a pharmacogenetic approach [39,62]. Varenicline, a partial agonist of nicotinic acetylcholine receptors, has shown variability in its efficacy according to the patient’s genetic profile [63]. Genes of particular interest in this regard include the *OCT2* transporter (*SLC22A2*), which transports both nicotine and varenicline [64]; the nicotinic acetylcholine receptor genes *CHRNA4* [65] and *CHRNB2* [65]; the *CHRNA5*-*CHRNA3*-*CHRNB4* gene cluster [29,66]; and the *CYP2A6* [67] and *CYP2B6* [68] genes, which are involved in nicotine metabolism. In addition to efficacy, the safety of varenicline also appears to be influenced by genetic factors. Thus, a very recent GWAS identified several variants in the *ICAM5* gene that are associated with the occurrence of adverse effects (unusual nightmares) in patients treated with this drug [63].

Similarly, the effectiveness of bupropion, which acts by modulating the dopaminergic system, is influenced by variants in genes such as *CYP2B6* [65,69], which metabolically activates the drug by converting it into hydroxybupropion, *CHRNB2* [70], and other genes related to dopamine neurotransmission. With regard to the latter, David et al. showed that the Taq1A variant of the dopamine D2 receptor was associated with treatment efficacy [71]. The same research group developed a score based on variants present in genes involved in dopaminergic pathways, such as COMT (dopamine metabolism), DRD4 (dopamine receptor), and SLC6A3 (dopamine reuptake transporter), which was able to predict the duration of abstinence following a smoking cessation attempt treated with bupropion [72]. Finally, an association has also been reported between abstinence during bupropion treatment and genetic variability in the serotonin transporter *SLC6A4*, probably owing to a possible interaction between the drug and serotonergic receptors [73].

Regarding cytisinicline, given its recent introduction into smoking cessation therapies, there are still limited data suggesting an influence of genetic factors on its efficacy or safety. However, a recent study linked some of its effects to the expression of the serotonin transporter, which in turn could influence nicotine dependence [74]. Finally, *CYP2A6* variants may also influence the optimal dose of nicotine replacement therapies. Malaiyandi et al. demonstrated that the *CYP2A6* genotype influences the plasma nicotine concentrations achieved during these therapies. The authors suggested that prior genetic testing could be useful for predicting these concentrations and adjusting doses accordingly [40]. Likewise, Sarginson et al. found an association between the rs680244 variant of *CHRNA5* and long-term abstinence in patients receiving combined treatment with bupropion and nicotine patches [75]. In contrast, a meta-analysis of patients receiving nicotine replacement therapy found no association between treatment effectiveness and the presence of variants in the *CHRNA5*-*CHRNA3*-*CHRNB4* gene cluster [76].

Genetic knowledge is also guiding the development of new therapies specifically targeting pathways involved in TUD. Zeilinger et al. reported that tobacco smoking induces profound epigenetic changes at the level of DNA methylation [52]. These alterations have also prompted interest in whether epigenetic mechanisms could represent therapeutic targets, as, unlike permanent alterations in the DNA sequence, many epigenetic marks are potentially reversible, providing a rationale for the development of so-called “epidrugs”, which include DNA methyltransferase (DNMTs) inhibitors, histone-modifying enzymes or bromodomain and extra-terminal (BET) inhibitors. [77]. Notwithstanding, epigenetic regulation is highly tissue-, cell-, and context-dependent, and broad pharmacological manipulation of the epigenome may produce unintended effects. Therefore, although the concept of a potentially “curable” or reversible epigenome is attractive, the therapeutic application of epidrugs to nicotine dependence remains theoretical, and further evidence is required. In addition, preclinical studies are also exploring the development of molecules that selectively modulate the activity of nicotinic receptors altered by genetic variants. For example, pozanicline (ABT-089) and related compounds are partial agonists acting on these receptors, and their use in experimental animals has been shown to alleviate nicotine withdrawal symptoms [78].

However, the success of smoking cessation treatment depends not only on pharmacotherapy but also on behavioral interventions, which could likewise be personalized by considering the patient’s genetic profile. For example, based on the results of several GWAS, Sallis et al. found evidence of genetic correlations between certain personality traits and tobacco-related phenotypes, and suggested that these findings could be used to personalize future smoking cessation interventions and identify those patients who are most susceptible to relapse [79].

**Figure 3:**
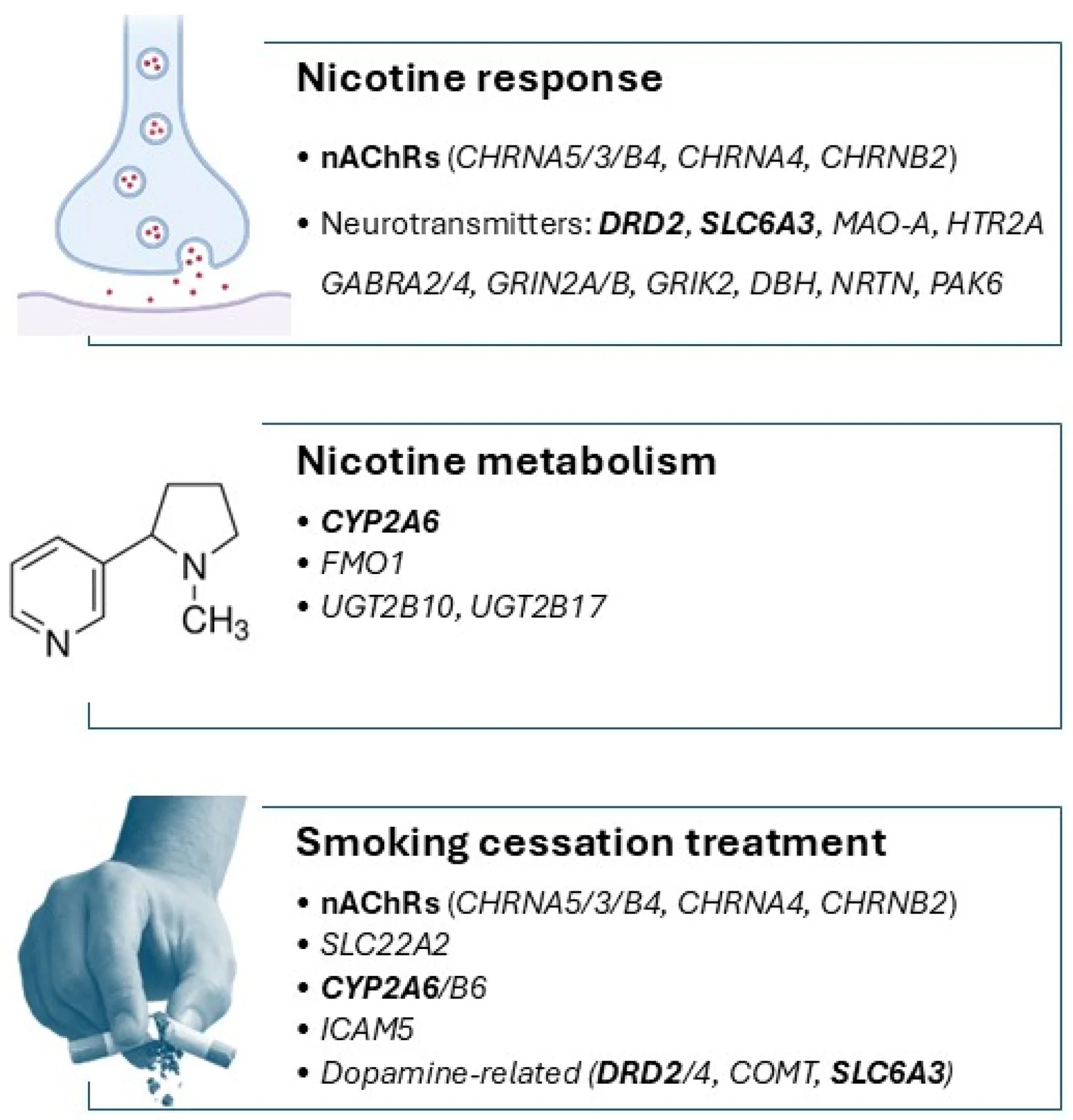
Genetic variation may influence tobacco use disorder through three interconnected domains treated in this review: (1) neuronal and neurotransmitter pathways mediating the response to nicotine; (2) enzymatic pathways controlling nicotine metabolism and clearance; and (3) pharmacogenetic mechanisms influencing the response to tobacco-use disorder treatments. Highlighted genes participate in more than one of these domains. nAChRs, nicotinic acetylcholine receptors.

See Table 2 for a summary of the most relevant findings regarding the genetics of cessation therapy. In addition, Figure 3 shows a diagram of genes involved in these drugs’ pharmacogenetics, along with those with a reported influence on nicotine metabolism and response to the alkaloid, the three aspects of smoking genetics covered in this review.

## 5. Knowledge Gaps and Current Limitations

Despite substantial progress in understanding the genetic basis of TUD, several important knowledge gaps remain. First, the complete genetic architecture of TUD has not yet been elucidated. Although large-scale GWAS have identified numerous susceptibility loci, the proportion of phenotypic variance explained by these variants remains lower than estimated heritability, indicating that a substantial component of genetic susceptibility remains unexplained. The relative contribution of common and rare variants also remains incompletely characterized. Moreover, smoking initiation, smoking intensity, persistence, nicotine dependence, and cessation are related but distinct phenotypes, and the extent to which they share common genetic determinants is not fully established. A second limitation concerns the biological interpretation of genetic associations. For many GWAS loci, the associated variant does not necessarily represent the causal variant, and the functional gene and cellular mechanisms underlying the association remain uncertain. Third, as discussed earlier, the generalizability of genetic findings across populations also remains limited, with an incomplete representation of global genetic diversity, which hampers, for instance, the reproducibility of PRS. Finally, the clinical relevance of genetic findings remains incompletely established. Indeed, although genetic variants and PRS have been associated with smoking susceptibility and treatment response, their additional predictive value beyond established clinical and behavioral factors has not been consistently demonstrated, as discussed in the next section.

## 6. Perspectives and Conclusions

Research on the genetic basis of TUD has advanced considerably, opening new opportunities in both the clinical and preventive fields. Candidate gene association studies, but above all GWAS and PRS, among other approaches, have made it possible to identify critical genetic pathways involved in the effects of tobacco. In this regard, the integration of artificial intelligence into genomic studies will, in the short to medium term, enable the analysis of complex interactions between genetic variants, environmental exposures, and treatments, thereby accelerating the personalization of therapies and facilitating the development of predictive algorithms [80].

The identification of genetic associations represents an important first step, but the biological interpretation of associated loci requires complementary functional approaches. Integration of GWAS results with eQTL and transcriptomic data can help identify the genes whose expression is affected by risk variants, while chromatin accessibility and other epigenomic approaches can reveal regulatory elements and cell types in which these variants exert their effects. Single-cell sequencing further provides cell-type-specific resolution, which is particularly relevant for complex phenotypes such as nicotine dependence involving multiple brain circuits and neuronal populations. In addition, genome-editing approaches such as CRISPR can experimentally test the functional consequences of candidate variants or regulatory regions, helping to distinguish causal mechanisms from statistical associations. For instance, integration of GWAS with GTEx eQTL data and H-MAGMA/S-MultiXcan analyses has implicated specific genes at established nicotine-dependence loci [81]. In another example, eQTL analyses of the *CHRNA5–CHRNA3–CHRNB4* cluster have further shown that regulatory variants influence gene expression and modify nicotine-dependence risk, illustrating how functional genomics can bridge the gap between genetic association and biological mechanism [82].

One of the most promising areas for the application of these genetic findings is the development of personalized medicine strategies for smoking cessation therapies. It has been shown that the drugs currently approved for this purpose in Spain, particularly varenicline and bupropion, may exhibit reduced effectiveness owing to the presence of genetic variants. This opens the possibility of performing genetic testing before treatment in order to determine the most appropriate drug and dosage for each patient. However, smoking cessation therapies are not the only area that could benefit from precision medicine. Genetic information could also be incorporated into educational programs and public health policies to discourage smoking initiation among genetically susceptible individuals [83], or to identify populations with a greater genetic susceptibility to developing tobacco-related diseases, thereby facilitating the implementation of early prevention programs [58,84].

However, despite these promising advances, important barriers remain to the widespread implementation of personalized medicine strategies. These include unequal access to genetic testing depending on the healthcare setting, the fact that the technologies required to identify genetic profiles are not yet economically feasible for many healthcare systems, and concerns regarding privacy, since knowledge of an individual’s genetic risk could lead to stigmatization or discourage people from seeking medical assistance [85]. In the future, combining multidisciplinary approaches ranging from genetics to social sciences will be essential to overcome these barriers and transform tobacco control into a precision medicine model, ultimately improving quality of life and reducing the impact of this global epidemic on public health.

## Figures and Tables

**Table 2:** Influence of genetics on the effectiveness and toxicity of drugs used in smoking cessation treatments.

Reference	Drug	Affected Pathway	Gene(s) and Variant(s)	Comment
Bergen et al., 2014 [64]	Varenicline	Drug transport	*SLC22A2* (*OCT2*) rs316019	Variant associated with a higher abstinence rate in European populations.
King et al., 2012 [65]	Varenicline	Nicotinic acetylcholine receptors	*CHRNA4* rs3787138, *CHRNB2* rs3811450	Variants associated with therapeutic response.
Lassi et al., 2016 [29]	Varenicline	Nicotinic acetylcholine receptors	*CHRNA5-CHRNA3-CHRNB4* gene cluster	Genetic variability within the cluster influences clinical response.
Chen et al., 2020 [66]	Varenicline	Nicotinic acetylcholine receptors	*CHRNA5* rs16969968, rs680244	Carriers showed reduced treatment efficacy.
Chenoweth et al., 2023 [67]	Varenicline	Nicotine metabolism	*CYP2A6* loss-of-function alleles	Slow nicotine metabolism influences treatment success.
Tomaz et al., 2019 [68]	Varenicline	Nicotine metabolism	*CYP2B6* rs8109525	Polymorphism associated with successful treatment.
Chenoweth et al., 2024 [63]	Varenicline	Hippocampal and cortical neuronal function	*ICAM5* rs901886	First GWAS identifying variants associated with varenicline-induced nightmares.
Zhu et al., 2012 [69]	Bupropion	Drug metabolism	*CYP2B6* loss-of-function alleles	Carriers require higher drug doses to achieve adequate hydroxybupropion concentrations.
Conti et al., 2008 [70]	Bupropion	Nicotinic acetylcholine receptors	*CHRNB2* rs2072661	Carriers of the A allele showed lower treatment effectiveness.
King et al., 2012 [65]	Bupropion	Drug metabolism	*CYP2B6* rs8109525, rs1808682	Variants associated with sustained abstinence.
David et al., 2007 [71]	Bupropion	Dopaminergic system	*DRD2* rs1800497 (Taq1A)	Variant associated with higher abstinence rates.
David et al., 2013 [72]	Bupropion	Dopaminergic system	*COMT*, *DRD4*, *SLC6A3* (genetic score)	The dopaminergic genetic score predicts smoking abstinence.
Verde et al., 2014 [73]	Bupropion	Serotonergic system	*SLC6A4* 5-HTTLPR, *HTR2A* A-1438G	Association between serotonergic variants and treatment success.
Mineur et al., 2015 [74]	Cytisinicline	Serotonergic system	*HTR1A*	Receptor expression may modulate the antidepressant effects of the drug.
Malaiyandi et al., 2006 [40]	Nicotine patches	Nicotine metabolism	*CYP2A6* loss-of-function alleles	Higher plasma nicotine concentrations with the same number of nicotine patches.
Sarginson et al., 2011 [75]	Nicotine patches + Bupropion	Nicotinic acetylcholine receptors	*CHRNA5* rs680244	Variant associated with long-term abstinence.
Leung et al., 2015 [76]	Nicotine patches	Nicotinic acetylcholine receptors	*CHRNA5-CHRNA3-CHRNB4* gene cluster	No association between genetic variability within the cluster and treatment effectiveness.

## Data Availability

No new data were created or analyzed in this study. Data sharing is not applicable to this article.

## References

[1] WHO Tobacco and Nicotine. https://www.who.int/news-room/fact-sheets/detail/tobacco.

[2] Gallucci G., Tartarone A., Lerose R., Lalinga A.V., Capobianco A.M. (2020). Cardiovascular risk of smoking and benefits of smoking cessation. J. Thorac. Dis..

[3] Changeux J.P. (2010). Nicotine addiction and nicotinic receptors: Lessons from genetically modified mice. Nat. Rev. Neurosci..

[4] Picciotto M.R., Caldarone B.J., King S.L., Zachariou V. (2000). Nicotinic receptors in the brain. Links between molecular biology and behavior. Neuropsychopharmacology.

[5] Jiloha R.C. (2010). Biological basis of tobacco addiction: Implications for smoking-cessation treatment. Indian J. Psychiatry.

[6] (2008). Clinical Practice Guideline Treating Tobacco Use and Dependence 2008 Update Panel, Liaisons, and Staff. A clinical practice guideline for treating tobacco use and dependence: 2008 update. A U.S. Public Health Service report. Am. J. Prev. Med..

[7] Loukola A., Hallfors J., Korhonen T., Kaprio J. (2014). Genetics and smoking. Curr. Addict. Rep..

[8] Carmelli D., Swan G.E., Robinette D., Fabsitz R. (1992). Genetic influence on smoking—A study of male twins. N. Engl. J. Med..

[9] Heath A.C., Cates R., Martin N.G., Meyer J., Hewitt J.K., Neale M.C., Eaves L.J. (1993). Genetic contribution to risk of smoking initiation: Comparisons across birth cohorts and across cultures. J. Subst. Abus..

[10] Heath A.C., Martin N.G. (1993). Genetic models for the natural history of smoking: Evidence for a genetic influence on smoking persistence. Addict. Behav..

[11] Agrawal A., Madden P.A., Bucholz K.K., Heath A.C., Lynskey M.T. (2014). Initial reactions to tobacco and cannabis smoking: A twin study. Addiction.

[12] Li M.D., Cheng R., Ma J.Z., Swan G.E. (2003). A meta-analysis of estimated genetic and environmental effects on smoking behavior in male and female adult twins. Addiction.

[13] Thorgeirsson T.E., Geller F., Sulem P., Rafnar T., Wiste A., Magnusson K.P., Manolescu A., Thorleifsson G., Stefansson H., Ingason A. (2008). A variant associated with nicotine dependence, lung cancer and peripheral arterial disease. Nature.

[14] Caporaso N., Gu F., Chatterjee N., Sheng-Chih J., Yu K., Yeager M., Chen C., Jacobs K., Wheeler W., Landi M.T. (2009). Genome-wide and candidate gene association study of cigarette smoking behaviors. PLoS ONE.

[15] Liu J.Z., Tozzi F., Waterworth D.M., Pillai S.G., Muglia P., Middleton L., Berrettini W., Knouff C.W., Yuan X., Waeber G. (2010). Meta-analysis and imputation refines the association of 15q25 with smoking quantity. Nat. Genet..

[16] Tobacco and Genetics Consortium (2010). Genome-wide meta-analyses identify multiple loci associated with smoking behavior. Nat. Genet..

[17] David S.P., Hamidovic A., Chen G.K., Bergen A.W., Wessel J., Kasberger J.L., Brown W.M., Petruzella S., Thacker E.L., Kim Y. (2012). Genome-wide meta-analyses of smoking behaviors in African Americans. Transl. Psychiatry.

[18] Yoon D., Kim Y.J., Cui W.Y., Van der Vaart A., Cho Y.S., Lee J.Y., Ma J.Z., Payne T.J., Li M.D., Park T. (2012). Large-scale genome-wide association study of Asian population reveals genetic factors in FRMD4A and other loci influencing smoking initiation and nicotine dependence. Hum. Genet..

[19] Matoba N., Akiyama M., Ishigaki K., Kanai M., Takahashi A., Momozawa Y., Ikegawa S., Ikeda M., Iwata N., Hirata M. (2019). GWAS of smoking behaviour in 165,436 Japanese people reveals seven new loci and shared genetic architecture. Nat. Hum. Behav..

[20] Vink J.M., Posthuma D., Neale M.C., Eline Slagboom P., Boomsma D.I. (2006). Genome-wide linkage scan to identify Loci for age at first cigarette in Dutch sibling pairs. Behav. Genet..

[21] Erzurumluoglu A.M., Liu M., Jackson V.E., Barnes D.R., Datta G., Melbourne C.A., Young R., Batini C., Surendran P., Jiang T. (2020). Meta-analysis of up to 622,409 individuals identifies 40 novel smoking behaviour associated genetic loci. Mol. Psychiatry.

[22] Xu K., Li B., McGinnis K.A., Vickers-Smith R., Dao C., Sun N., Kember R.L., Zhou H., Becker W.C., Gelernter J. (2020). Genome-wide association study of smoking trajectory and meta-analysis of smoking status in 842,000 individuals. Nat. Commun..

[23] Saunders G.R.B., Wang X., Chen F., Jang S.K., Liu M., Wang C., Gao S., Jiang Y., Khunsriraksakul C., Otto J.M. (2022). Genetic diversity fuels gene discovery for tobacco and alcohol use. Nature.

[24] Vink J.M., Willemsen G., Boomsma D.I. (2005). Heritability of smoking initiation and nicotine dependence. Behav. Genet..

[25] Evans L.M., Jang S., Hancock D.B., Ehringer M.A., Otto J.M., Vrieze S.I., Keller M.C. (2021). Genetic architecture of four smoking behaviors using partitioned SNP heritability. Addiction.

[26] Deak J.D., Clark D.A., Liu M., Schaefer J.D., Jang S.K., Durbin C.E., Iacono W.G., McGue M., Vrieze S., Hicks B.M. (2022). Alcohol and nicotine polygenic scores are associated with the development of alcohol and nicotine use problems from adolescence to young adulthood. Addiction.

[27] Bray M.J., Chen L.S., Fox L., Ma Y., Grucza R.A., Hartz S.M., Culverhouse R.C., Saccone N.L., Hancock D.B., Johnson E.O. (2021). Studying the Utility of Using Genetics to Predict Smoking-Related Outcomes in a Population-Based Study and a Selected Cohort. Nicotine Tob. Res..

[28] Risner V.A., Benca-Bachman C.E., Bertin L., Smith A.K., Kaprio J., McGeary J.E., Chesler E., Knopik V.S., Friedman N.P., Palmer R.H.C. (2021). Multi-Polygenic Analysis of Nicotine Dependence in Individuals of European Ancestry. Nicotine Tob. Res..

[29] Lassi G., Taylor A.E., Timpson N.J., Kenny P.J., Mather R.J., Eisen T., Munafo M.R. (2016). The CHRNA5-A3-B4 Gene Cluster and Smoking: From Discovery to Therapeutics. Trends Neurosci..

[30] Greenbaum L., Kanyas K., Karni O., Merbl Y., Olender T., Horowitz A., Yakir A., Lancet D., Ben-Asher E., Lerer B. (2006). Why do young women smoke? I. Direct and interactive effects of environment, psychological characteristics and nicotinic cholinergic receptor genes. Mol. Psychiatry.

[31] Thorgeirsson T.E., Steinberg S., Reginsson G.W., Bjornsdottir G., Rafnar T., Jonsdottir I., Helgadottir A., Gretarsdottir S., Helgadottir H., Jonsson S. (2016). A rare missense mutation in CHRNA4 associates with smoking behavior and its consequences. Mol. Psychiatry.

[32] Munafo M., Clark T., Johnstone E., Murphy M., Walton R. (2004). The genetic basis for smoking behavior: A systematic review and meta-analysis. Nicotine Tob. Res..

[33] Jonsson E.G., Nothen M.M., Grunhage F., Farde L., Nakashima Y., Propping P., Sedvall G.C. (1999). Polymorphisms in the dopamine D2 receptor gene and their relationships to striatal dopamine receptor density of healthy volunteers. Mol. Psychiatry.

[34] Ma Y., Wang M., Yuan W., Su K., Li M.D. (2015). The significant association of Taq1A genotypes in DRD2/ANKK1 with smoking cessation in a large-scale meta-analysis of Caucasian populations. Transl. Psychiatry.

[35] Tiili E.M., Mitiushkina N.V., Sukhovskaya O.A., Imyanitov E.N., Hirvonen A.P. (2020). The effect of SLC6A3 variable number of tandem repeats and methylation levels on individual susceptibility to start tobacco smoking and on the ability of smokers to quit smoking. Pharmacogenet. Genom..

[36] McKinney E.F., Walton R.T., Yudkin P., Fuller A., Haldar N.A., Mant D., Murphy M., Welsh K.I., Marshall S.E. (2000). Association between polymorphisms in dopamine metabolic enzymes and tobacco consumption in smokers. Pharmacogenetics.

[37] do Prado-Lima P.A., Chatkin J.M., Taufer M., Oliveira G., Silveira E., Neto C.A., Haggstram F., Bodanese L.C., da Cruz I.B. (2004). Polymorphism of 5HT2A serotonin receptor gene is implicated in smoking addiction. Am. J. Med. Genet. B Neuropsychiatr. Genet..

[38] Dempsey D., Tutka P., Jacob P., Allen F., Schoedel K., Tyndale R.F., Benowitz N.L. (2004). Nicotine metabolite ratio as an index of cytochrome P450 2A6 metabolic activity. Clin. Pharmacol. Ther..

[39] El-Boraie A., Tyndale R.F. (2021). The Role of Pharmacogenetics in Smoking. Clin. Pharmacol. Ther..

[40] Malaiyandi V., Lerman C., Benowitz N.L., Jepson C., Patterson F., Tyndale R.F. (2006). Impact of CYP2A6 genotype on pretreatment smoking behaviour and nicotine levels from and usage of nicotine replacement therapy. Mol. Psychiatry.

[41] Chenoweth M.J., Tyndale R.F. (2017). Pharmacogenetic Optimization of Smoking Cessation Treatment. Trends Pharmacol. Sci..

[42] Buchwald J., Chenoweth M.J., Palviainen T., Zhu G., Benner C., Gordon S., Korhonen T., Ripatti S., Madden P.A.F., Lehtimaki T. (2021). Genome-wide association meta-analysis of nicotine metabolism and cigarette consumption measures in smokers of European descent. Mol. Psychiatry.

[43] Bloom A.J., Baker T.B., Chen L.S., Breslau N., Hatsukami D., Bierut L.J., Goate A. (2014). Variants in two adjacent genes, EGLN2 and CYP2A6, influence smoking behavior related to disease risk via different mechanisms. Hum. Mol. Genet..

[44] Agrawal A., Pergadia M.L., Balasubramanian S., Saccone S.F., Hinrichs A.L., Saccone N.L., Breslau N., Johnson E.O., Hatsukami D., Martin N.G. (2009). Further evidence for an association between the gamma-aminobutyric acid receptor A, subunit 4 genes on chromosome 4 and Fagerstrom Test for Nicotine Dependence. Addiction.

[45] Scofield M.D., Heinsbroek J.A., Gipson C.D., Kupchik Y.M., Spencer S., Smith A.C., Roberts-Wolfe D., Kalivas P.W. (2016). The Nucleus Accumbens: Mechanisms of Addiction across Drug Classes Reflect the Importance of Glutamate Homeostasis. Pharmacol. Rev..

[46] Dawidowski B., Olejniczak B., Groblinska K., Knapinska M., Kozicka U., Krasinski M., Kulak A., Grelecki G., Czaplinska Z., Piotrowska O. (2024). The rs4354668 polymorphism in the SLC1A2 gene for the EAAT2 glutamate transporter is associated with an increased risk of harmful drug use—An exploratory study on a university student population. Psychiatr. Pol..

[47] Vink J.M., Smit A.B., de Geus E.J., Sullivan P., Willemsen G., Hottenga J.J., Smit J.H., Hoogendijk W.J., Zitman F.G., Peltonen L. (2009). Genome-wide association study of smoking initiation and current smoking. Am. J. Hum. Genet..

[48] Perez-Paramo Y.X., Watson C.J.W., Chen G., Thomas C.E., Adams-Haduch J., Wang R., Khor C.C., Koh W.P., Nelson H.H., Yuan J.M. (2023). Impact of Genetic Variants in the Nicotine Metabolism Pathway on Nicotine Metabolite Levels in Smokers. Cancer Epidemiol. Biomark. Prev..

[49] Zhang T.X., Saccone N.L., Bierut L.J., Rice J.P. (2017). Targeted sequencing identifies genetic polymorphisms of flavin-containing monooxygenase genes contributing to susceptibility of nicotine dependence in European American and African American. Brain Behav..

[50] Ware J.J., Chen X., Vink J., Loukola A., Minica C., Pool R., Milaneschi Y., Mangino M., Menni C., Chen J. (2016). Genome-Wide Meta-Analysis of Cotinine Levels in Cigarette Smokers Identifies Locus at 4q13.2. Sci. Rep..

[51] Loukola A., Wedenoja J., Keskitalo-Vuokko K., Broms U., Korhonen T., Ripatti S., Sarin A.P., Pitkaniemi J., He L., Happola A. (2014). Genome-wide association study on detailed profiles of smoking behavior and nicotine dependence in a twin sample. Mol. Psychiatry.

[52] Zeilinger S., Kuhnel B., Klopp N., Baurecht H., Kleinschmidt A., Gieger C., Weidinger S., Lattka E., Adamski J., Peters A. (2013). Tobacco smoking leads to extensive genome-wide changes in DNA methylation. PLoS ONE.

[53] Collins F.S. (1999). Shattuck lecture—Medical and societal consequences of the Human Genome Project. N. Engl. J. Med..

[54] Hao L., Kraft P., Berriz G.F., Hynes E.D., Koch C., Korategere V.K.P., Parpattedar S.S., Steeves M., Yu W., Antwi A.A. (2022). Development of a clinical polygenic risk score assay and reporting workflow. Nat. Med..

[55] Foo J.C., Volker M.P., Streit F., Frank J., Zacharias N., Zillich L., Sirignano L., Nurnberg P., Wienker T.F., Wagner M. (2024). Polygenic risk scores for nicotine use and family history of smoking are associated with smoking behaviour. Drug Alcohol. Depend..

[56] Acosta J.N., Szejko N., Both C.P., Vanent K., Noche R.B., Gill T.M., Matouk C.C., Sheth K.N., Gunel M., Falcone G.J. (2021). Genetically Determined Smoking Behavior and Risk of Nontraumatic Subarachnoid Hemorrhage. Stroke.

[57] Ohi K., Nishizawa D., Muto Y., Sugiyama S., Hasegawa J., Soda M., Kitaichi K., Hashimoto R., Shioiri T., Ikeda K. (2020). Polygenic risk scores for late smoking initiation associated with the risk of schizophrenia. npj Schizophr..

[58] Jia G., Wen W., Massion P.P., Shu X.O., Zheng W. (2021). Incorporating both genetic and tobacco smoking data to identify high-risk smokers for lung cancer screening. Carcinogenesis.

[59] Gordon L.G., Hirst N.G., Young R.P., Brown P.M. (2010). Within a smoking-cessation program, what impact does genetic information on lung cancer need to have to demonstrate cost-effectiveness?. Cost. Eff. Resour. Alloc..

[60] Bray M., Chang Y., Baker T.B., Jorenby D., Carney R.M., Fox L., Pham G., Stoneking F., Smock N., Amos C.I. (2022). The Promise of Polygenic Risk Prediction in Smoking Cessation: Evidence from Two Treatment Trials. Nicotine Tob. Res..

[61] Stevens V.L., Jacobs E.J., Gapstur S.M., Carter B.D., Gaudet M.M., Westmaas J.L., Patel A.V. (2017). Evaluation of a Novel Difficulty of Smoking Cessation Phenotype Based on Number of Quit Attempts. Nicotine Tob. Res..

[62] Lerman C.E., Schnoll R.A., Munafo M.R. (2007). Genetics and smoking cessation improving outcomes in smokers at risk. Am. J. Prev. Med..

[63] Chenoweth M.J., Kim Y.J., Nollen N.L., Hawk L.W., Mahoney M.C., Lerman C., Knight J., Tyndale R.F. (2024). Genetic Prediction of Smoking Cessation Medication Side Effects: A Genome-Wide Investigation of Abnormal Dreams on Varenicline. Clin. Pharmacol. Ther..

[64] Bergen A.W., Javitz H.S., Krasnow R., Michel M., Nishita D., Conti D.V., Edlund C.K., Kwok P.Y., McClure J.B., Kim R.B. (2014). Organic cation transporter variation and response to smoking cessation therapies. Nicotine Tob. Res..

[65] King D.P., Paciga S., Pickering E., Benowitz N.L., Bierut L.J., Conti D.V., Kaprio J., Lerman C., Park P.W. (2012). Smoking cessation pharmacogenetics: Analysis of varenicline and bupropion in placebo-controlled clinical trials. Neuropsychopharmacology.

[66] Chen L.S., Baker T.B., Miller J.P., Bray M., Smock N., Chen J., Stoneking F., Culverhouse R.C., Saccone N.L., Amos C.I. (2020). Genetic Variant in CHRNA5 and Response to Varenicline and Combination Nicotine Replacement in a Randomized Placebo-Controlled Trial. Clin. Pharmacol. Ther..

[67] Chenoweth M.J., Lerman C., Knight J., Tyndale R.F. (2023). Influence of CYP2A6 Genetic Variation, Nicotine Dependence Severity, and Treatment on Smoking Cessation Success. Nicotine Tob. Res..

[68] Tomaz P.R.X., Kajita M.S., Santos J.R., Scholz J., Abe T.O., Gaya P.V., Krieger J.E., Pereira A.C., Santos P. (2019). Cytochrome P450 2A6 and 2B6 polymorphisms and smoking cessation success in patients treated with varenicline. Eur. J. Clin. Pharmacol..

[69] Zhu A.Z., Cox L.S., Nollen N., Faseru B., Okuyemi K.S., Ahluwalia J.S., Benowitz N.L., Tyndale R.F. (2012). CYP2B6 and bupropion’s smoking-cessation pharmacology: The role of hydroxybupropion. Clin. Pharmacol. Ther..

[70] Conti D.V., Lee W., Li D., Liu J., Van Den Berg D., Thomas P.D., Bergen A.W., Swan G.E., Tyndale R.F., Benowitz N.L. (2008). Nicotinic acetylcholine receptor beta2 subunit gene implicated in a systems-based candidate gene study of smoking cessation. Hum. Mol. Genet..

[71] David S.P., Brown R.A., Papandonatos G.D., Kahler C.W., Lloyd-Richardson E.E., Munafo M.R., Shields P.G., Lerman C., Strong D., McCaffery J. (2007). Pharmacogenetic clinical trial of sustained-release bupropion for smoking cessation. Nicotine Tob. Res..

[72] David S.P., Strong D.R., Leventhal A.M., Lancaster M.A., McGeary J.E., Munafo M.R., Bergen A.W., Swan G.E., Benowitz N.L., Tyndale R.F. (2013). Influence of a dopamine pathway additive genetic efficacy score on smoking cessation: Results from two randomized clinical trials of bupropion. Addiction.

[73] Verde Z., Santiago C., Rodriguez Gonzalez-Moro J.M., de Lucas Ramos P., Lopez Martin S., Bandres F., Lucia A., Gomez-Gallego F. (2014). Are serotonergic system genes associated to smoking cessation therapy success in addition to CYP2A6?. Pharmacopsychiatry.

[74] Mineur Y.S., Einstein E.B., Bentham M.P., Wigestrand M.B., Blakeman S., Newbold S.A., Picciotto M.R. (2015). Expression of the 5-HT1A serotonin receptor in the hippocampus is required for social stress resilience and the antidepressant-like effects induced by the nicotinic partial agonist cytisine. Neuropsychopharmacology.

[75] Sarginson J.E., Killen J.D., Lazzeroni L.C., Fortmann S.P., Ryan H.S., Schatzberg A.F., Murphy G.M. (2011). Markers in the 15q24 nicotinic receptor subunit gene cluster (CHRNA5-A3-B4) predict severity of nicotine addiction and response to smoking cessation therapy. Am. J. Med. Genet. B Neuropsychiatr. Genet..

[76] Leung T., Bergen A., Munafo M.R., De Ruyck K., Selby P., De Luca V. (2015). Effect of the rs1051730-rs16969968 variant and smoking cessation treatment: A meta-analysis. Pharmacogenomics.

[77] Yano N., Fedulov A.V. (2023). Targeted DNA Demethylation: Vectors, Effectors and Perspectives. Biomedicines.

[78] Rollema H., Hurst R.S. (2018). The contribution of agonist and antagonist activities of alpha4beta2* nAChR ligands to smoking cessation efficacy: A quantitative analysis of literature data. Psychopharmacology.

[79] Sallis H.M., Davey Smith G., Munafo M.R. (2019). Cigarette smoking and personality: Interrogating causality using Mendelian randomisation. Psychol. Med..

[80] Faust I., Warden M., Camacho-Soto A., Racette B.A., Searles Nielsen S. (2023). A predictive algorithm to identify ever smoking in medical claims-based epidemiologic studies. Ann. Epidemiol..

[81] Quach B.C., Bray M.J., Gaddis N.C., Liu M., Palviainen T., Minica C.C., Zellers S., Sherva R., Aliev F., Nothnagel M. (2020). Expanding the genetic architecture of nicotine dependence and its shared genetics with multiple traits. Nat. Commun..

[82] Lee S.H., Ahn W.Y., Seweryn M., Sadee W. (2018). Combined genetic influence of the nicotinic receptor gene cluster CHRNA5/A3/B4 on nicotine dependence. BMC Genom..

[83] Lerman C. (2006). Helping smokers quit through pharmacogenetics. LDI Issue Brief..

[84] Wain L.V., Shrine N., Miller S., Jackson V.E., Ntalla I., Soler Artigas M., Billington C.K., Kheirallah A.K., Allen R., Cook J.P. (2015). Novel insights into the genetics of smoking behaviour, lung function, and chronic obstructive pulmonary disease (UK BiLEVE): A genetic association study in UK Biobank. Lancet Respir. Med..

[85] Clayton E.W., Halverson C.M., Sathe N.A., Malin B.A. (2018). A systematic literature review of individuals’ perspectives on privacy and genetic information in the United States. PLoS ONE.

